# Three-dimensional ultrasound assessment of endometrial receptivity: a review

**DOI:** 10.1186/1477-7827-4-56

**Published:** 2006-11-09

**Authors:** Juan Luis Alcázar

**Affiliations:** 1 Department of Obstetrics and Gynecology, Clínica Universitaria de Navarra, University of Navarra, Pamplona, Spain

## Abstract

Three-dimensional ultrasound (3D US) is a new imaging modality, which is being introduced into clinical practice. Although this technique will not probably replace two-dimensional ultrasound, it is being increasingly used. It has been reported that 3D US is a very high reproducible technique. The endometrium has been paid special attention when using this technique. The aim of this paper is to address some technical aspects of 3D US and to review critically its current status in evaluating endometrial function with special focus in its role in predicting pregnancy in assisted reproductive techniques. In spontaneous cycles endometrial volume grows during follicular phase remaining constant through the luteal phase. Endometrial vascularization increases during follicular phase peaking 2–3 days before ovulation, decreasing thereafter and increasing again during mid and late luteal phase. Data from studies analysing the role of 3D US for predicting IVF outcome are controversial. An explanation for these controversial findings might be different design of reported studies, specially the timing of ultrasound evaluation.

## Background

Endometrial receptivity is a crucial fact in human reproduction. Endometrial assessment has been performed usually by endometrial biopsy [1]. However, such as invasive method is not acceptable when evaluating endometrial receptivity in order not to damage the endometrium. Therefore, endometrial receptivity should be ideally evaluated before implantation by a non-invasive method.

Transvaginal ultrasonography may represent, theoretically, such an ideal non-invasive technique. Several parameters have been proposed for assessing endometrial receptivity, including endometrial thickness, endometrial pattern and endometrial and subendometrial blood flow [2-7]. These parameters may identify patients with low implantation potential. However, their positive predictive value is low [8,9].

Recently, three-dimensional ultrasound (3D US) has become available [10-13]. With this technology any desired plane through an organ can be obtained. With 3D US a volume of a region of interest (ROI) can be acquired and stored. This volume can be further analysed in several ways, such as navigation, multiplanar display, and surface rendering or volume calculation. This technique also allows a whole assessment of the endometrial and subendometrial vascularization [14,15]

In this review I shall address current state-of-the-art of 3D US in assessing the endometrium throughout the menstrual cycle and its possible role in predicting endometrial receptivity in assisted reproductive techniques (ARTs). A Medline search (1995–2006) was performed using the following key words: "three-dimensional ultrasound", "angiography", "power Doppler", "endometrium", "endometrial", "receptivity". A total of 27 articles were identified. Twenty-three were clinical studies and were selected for review, whereas 4 papers were reviews and were excluded.

## Technical aspects

Several published papers deal in detail the technical aspects of 3D US and an extensive description of these technical aspects is beyond the scope of this review [16-19]. Notwithstanding, I shall explain briefly some basic considerations.

3D US images can be obtained by two methods: freehand and automated. The freehand method requires manual movement of the transducer through the ROI. The automated method acquires the images using dedicated 3D transducers. When these probes are activated, the transducer elements automatically sweep through the ROI selected by the operator (the so-called "volume box") while the probe is held stationary. This provides more accuracy to this method as compared with the freehand systems, in which speed of sweep is more difficult to maintain constant manually by the operator.

**Figure 1:**
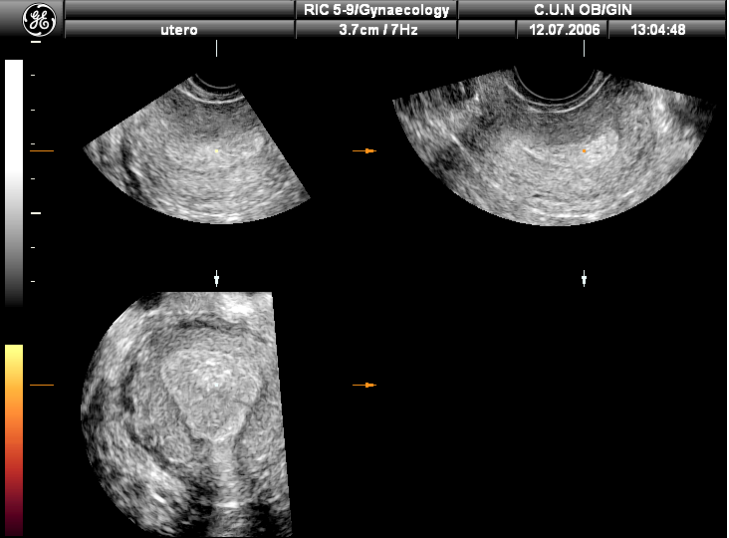
Three-dimensional ultrasound depicting multiplanar display of the uterus. All three orthogonal planes can be displayed using this technique.

The digitally stored volume data can be manipulated and presented in various displays: multiplanar display, "niche" mode or surface rendering mode. Probably, the most used and useful display is multiplanar display, which simultaneously shows three perpendicular planes (axial, sagital and coronal), allowing navigation through these three planes with the possibility of switch over any desired plane (Figure 1).

**Figure 2:**
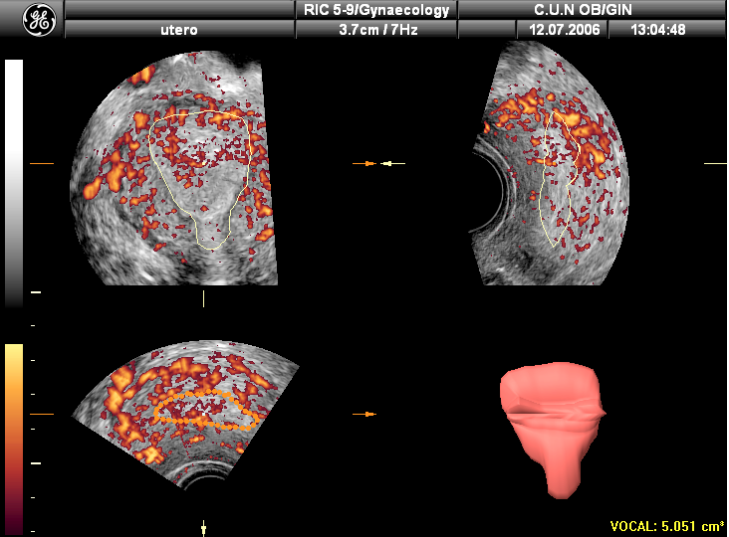
Endometrial volume calculation by using the VOCAL software after three-dimensional ultrasound.

Another important ability of 3D US is volume calculation, even in irregularly shaped structures, using the Virtual Organ Computer-aided AnaLysis (VOCAL) (Figure 2). This is a rotational method, based on rotations in given steps (6°, 9°, 15°, 30°) on a given orthogonal plane (A, B or C). This method has been demonstrated to be more accurate than 2D-volume estimation, with an error estimation of 7% for 3D US as compared of 22% for 2D US [17].

**Figure 3:**
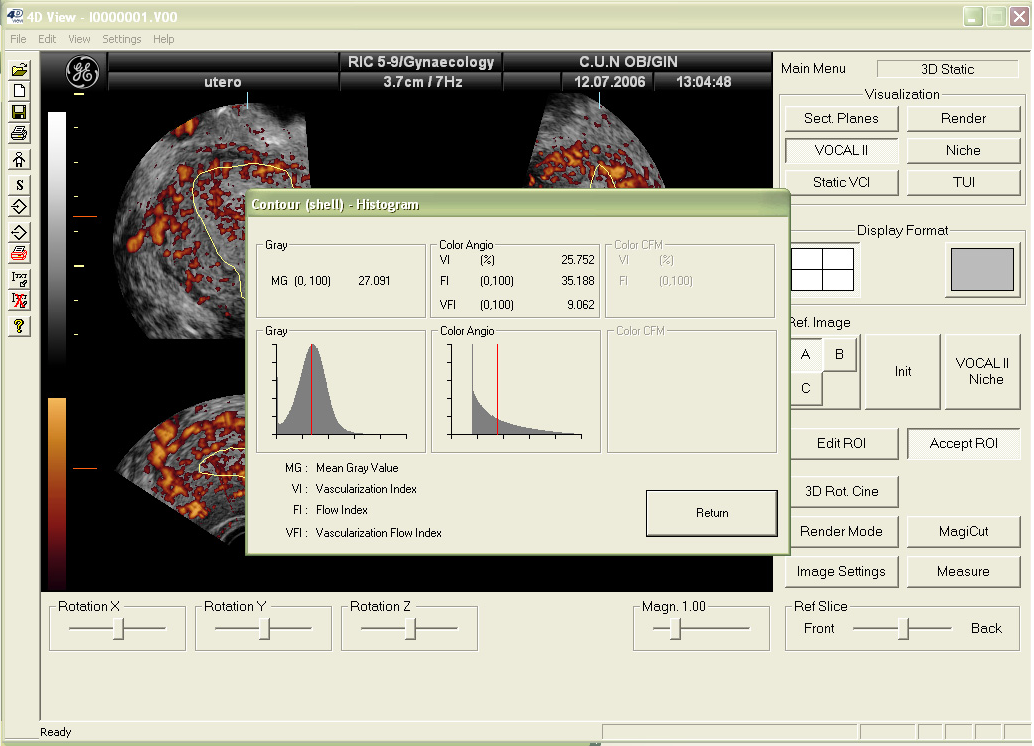
3D-Power Doppler indexes for assessing endometrial vascularization by means of the three-dimensional ultrasound.

**Figure 4:**
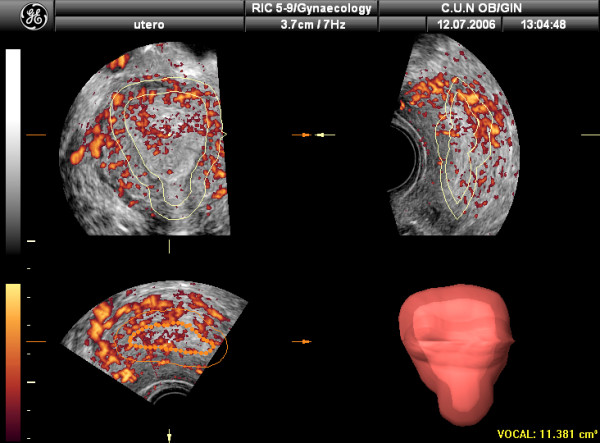
Determination of the subendometrial area volume by using the "shell" facility. In this case 5 mm has been chosen.

**Figure 5:**
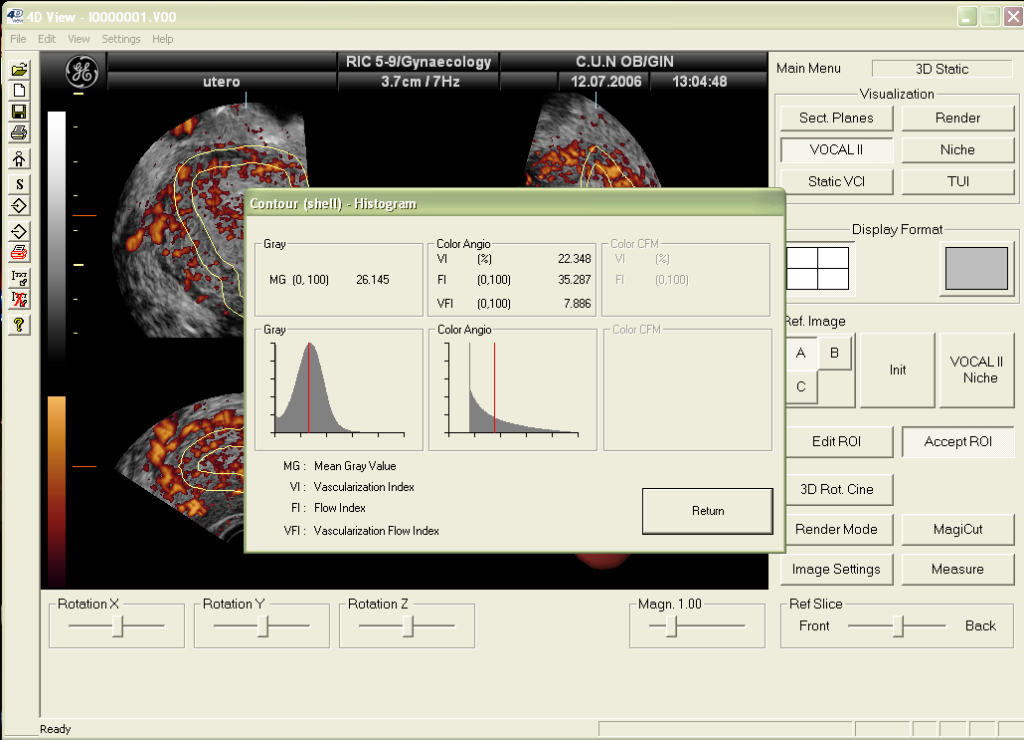
Vascularization of the subendometrial area by 3D-Power Doppler. VI, FI and VFI refers to the shell area, not the endometrium.

Vascularization of tissues within the ROI can be also assessed using 3D Power-Doppler ultrasound (3D-PDA) and the VOCAL program [18]. Using this method, three vascular indexes can be calculated: the Vascularization Index (VI), expressed as percentage, measures the number of colour voxels in the studied volume, representing the blood vessels within the tissue. The Flow Index (FI) is the average colour value of all colour voxels, representing average colour intensity. And the Vascular-Flow Index (VFI) is the average colour value of all grey and colour voxels, which represents both blood flow and vascularization (Figure 3). Using the "shell" function it is possible to calculate a volume at different thickness around the predetermined endometrium and estimate the vascularization in this "shell". This allows the assessment of the so-called "subendometrial region" (Figures 4 and 5)

3D US has a very low inter-observer and intra-observer variability for calculating endometrial volume, with intraclass correlation coefficients ≥ 0.97 [14,20-22]. However, this depends on the technique used, being the VOCAL method the most reproducible [23]. This technique has been also found to be highly reproducible for estimating ovarian and endometrial vascularization using 3D PD with intraclass correlation coefficients ≥ 0.99 for all indexes [15,24,25].

## Correlation of endometrial ultrasound and histology

Several studies have assessed the correlation between some endometrial sonographic parameters and histologic dating of the endometrium.

Li et al, using transabdominal ultrasonographic measurement of endometrial thickness prior to endometrial sampling in regularly cycling women found that endometrial histology was likely to be proliferative if the thickness was < 8 mm and likely to be secretory if endometrial thickness was ≥ 9 mm. However, for a given endometrial thickness, the stage of endometrial development appeared to vary widely, suggesting that ultrasonographic measurement of endometrial thickness cannot accurately predict histological dating [26]. These results have been confirmed by other studies [27,28].

Endometrial pattern, however, has been found to correlate with histologic dating of the endometrium [29-31]. Thus, a three-layered endometrium use to be present in the proliferative phase and an echogenic endometrium use to be present in the secretory phase [29].

Most studies evaluating the correlation of Doppler ultrasonographic assessment of uterine arteries and endometrial hisologic dating found that Doppler ultrasound cannot predict histologic dating [28,32].

To the best of my knowledge, no study has been published correlating 3D ultrasonographic data and histologic dating of the endometrium.

## Angiogenesis in the endometrium during the menstrual cycle and implantation

Controversy exists regarding angiogenesis, vascular density and expression of VEGF in the endometrium during normal menstrual cycle.

Some investigators have shown a significant increase in the vascular surface area, diameter and total number of capillaries in the secretory phase as compared with the proliferative phase [33]. Others have noted a peak in stromal VEGF expression in the proliferative phase with a peak glandular VEGF expression during the secretory phase [34].

Au and Rogers reported that angiogenesis was weakest during menstrual phase, followed by a rapid increase during the early proliferative phase to peak in mid-cycle before a gradually decrease towards cycle end [35].

Torry and Torry detected a significant increase in VEGF mRNA throughout the endometrial cycle in the non-pregnant patient with its expression increasing 3 to 5 times from the early proliferative phase to the late secretory phase [36].

On the contrary, some investigators have found that endometrial VEGF expression during the menstrual cycle is inconsistent [37] and others have reported no modifications in vascular density in different phases of the endometrial cycle [38] or that endothelial cell proliferation does not show a consistent pattern across the menstrual cycle [39]

Implantation is a progressive and versatile process in which the blastocyst apposes, attaches and invades the underlying endometrial surface. Angiogenesis is a crucial step fro embryo implantation. Several studies have demonstrated that VEGF and its receptors are markedly increased post ovulation and around peri-implantation period [40,41].

In summary, in spite of some controversial data, it seems that changes related to angiogenesis of reproduction are ovulatory-related and serve to prepare a receptive nidation site or the blastocyst/embryo [42-44].

## Three-dimensional evaluation of the endometrium in spontaneous menstrual cycles

Lee et al [45] first reported endometrial volume changes during spontaneous menstrual cycles assessed by 3D US. These authors reported on 18 nullipara regularly menstruating women, mean age 31 years. They performed a longitudinal study at 3–6 days interval during a single menstrual period, measuring the endometrial and uterine volume using the multi-slice technique and calculating the "uterus-endometrium" ratio. Mean endometrial volume was 1.23 cm^3 ^(SD: 0.98), ranging from 0.25 cm^3 ^to 5.5 cm^3^. They found that this ratio decreased throughout the menstrual cycle reaching a nadir around the 20^th ^cycle's day, reflecting that endometrial volume was highest at mid luteal phase (R^2 ^= 0.4318).

Raine-Fenning analysed the endometrial volume longitudinally in a series of 30 "apparently fertile" women, having regular menstrual cycle and no history of gynaecological disease [46]. Study design was an ultrasound examination n an alternate-day basis until ovulation, confirmed by ultrasound, and then every four days until next menstrual period. In this study the authors used the rotational method (plane C, rotation step 9°). This technique has been proven to be more precise and reproducible than multi-slice method for volume calculation [24]. They found a steady increase of the endometrial volume throughout the follicular phase until ovulation occurs and the remained relatively constant through luteal phase. As could be expected endometrial thickness was significantly correlated with endometrial volume (R^2 ^= 0.7671).

These findings would be in agreement with histological data in which endometrial growth is restricted to the follicular phase of the menstrual cycle when expansion of the stratum functionalis of the endometrium occurs, which in turns is directly related to the increase of serum estradiol levels. In this study endometrial volume was found to be greater in parous women. No relationship was found smoking or age.

This same group evaluated endometrial and subendometrial blood flow by 3D-PDA [47]. Subendometrial region was considered as an area within 5 mm of the originally defined myometrial-endometrial contour, using the "shell" software's facility. They found that both VI and VFI increased from mid-follicular phase, peaking 3 days prior to ovulation. Thereafter, there was a decrease in both of these indices, reaching a nadir 5 days postovulation, before a gradual increase during the transition from early to mid-luteal phase. FI showed a similar pattern but with a more pronounced nadir in late follicular phase. These changes in VI; FI and VFI were closely correlated with estradiol levels during the follicular phase but this relationship was lost after ovulation. All three indices began to rise when serum progesterone levels increased during luteal phase.

These findings were rather conflicting with data obtained from conventional pulsed Doppler studies in which uterine blood flow showed a steady increase throughout the menstrual cycle peaking in mid-luteal phase [48,49]. Most of these studies assume that blood flow within the uterine arteries is representative of the whole uterine and endometrial perfusion. However, power Doppler is more sensitive to lower velocity and combined with 3D US provides information from a specific region of the uterus (endometrial and subendometrial area). On the other hand, preovulatory reduction in 3D-PDA indices might be explained by a physical vessel obstruction induces by an increase in myometrial contractility [50].

More recently, Jokubkiene et al have reported similar findings on a group of 16 regular menstruating healthy women [51]. These researchers performed a prospective longitudinal assessment through the menstrual cycle on a daily basis from day 2, 3 or 4 until follicular rupture and then on days 1, 2, 5, 7 and 12 after ovulation. They used the VOCAL program (plane A, 30° rotation step). Subendometrial region was defined as 2 mm shell within the defined endometrial contour. Regarding endometrial volume their findings were identical to those from Raine-Fenning [47], an increase during the follicular phase and then plateaued throughout the luteal phase. In terms of vascularization, VI and VFI increased during the follicular phase reaching a maximum 2 days before ovulation, then decreased to reach a nadir 2 days after ovulation and then rose again progressively during the luteal phase. Changes in FI were similar but less clear, reaching the nadir 5 days after ovulation. However, these authors did not find a correlation between VI; FI and VFI in endometrial and subendometrial regions and progesterone levels on day +7 after ovulation or LH levels on days -1 or +1.

Different study design and methodology could explain differences between these two studies. Notwithstanding, in spite of these differences, both studies clearly show that changes in endometrial and subendometrial vascularization are ovulatory-related and would be in agreement with those previously mentioned studies that evaluated VEGF expression [34,36].

These studies are summarized in table 1.

**Table 1:** Summary of data published about the role of 3D-ultrasound for assessing normal menstrual cycle

Author	n	Primary Outcome	3D Method	Study Design	Findings
Lee (45)	18	Uterine-Endometrial volume ratio	Multislice	Longitudinal at 3–6 days interval	Uterine-Endometrial volume ratio decrease throughout menstrual cycle (R^2 ^= 0.4318)
Raine-Fenning (46)	30	Endometrial volume	VOCAL	Longitudinal at 2 days interval in follicular phase and 4 days interval in luteal phase	Endometrial volume increase steadily during follicular phase, plateauing during luteal phase
Raine-Fenning (47)	27	Endometrial and subendometrial vascularity	VOCAL	Longitudinal at 2 days interval in follicular phase and 4 days interval in luteal phase	Endometrial and subendometrial VI/FI/VFI increased from mid-follicular phase peaking 3 days prior to ovulation and then decrease until 5 days postovulation, increasing then again until the next cycle
Jokubkiene (50)	16	Endometrial volume, and endometrial and subendometrial vascularity	VOCAL	Longitudinal, daily in follicular phase and 2 days interval in luteal phase	Endometrial and subendometrial VI/FI/VFI increased from mid-follicular phase peaking 2 days prior to ovulation and then decrease until 2 days after ovulation, increasing then again until the next cycle

## Factors that may affect endometrial/subendometrial blood flow

Ng et al compared endometrial and subendometrial vascularization as assessed by 3D-PDA -rotational method, plane C, 15°-rotation step- and uterine artery blood flow by pulsed Doppler in spontaneous and stimulated cycles [52]. Subendometrial region was considered as 1-mm shell within the defined endometrial contour. Measurements were not longitudinally performed, but just once in hCG +2 day in stimulated cycles and in LH +1 day in spontaneous cycles. They found that endometrial volume was significantly greater in stimulated cycles as compared with spontaneous ones, whereas endometrial and subendometrial VI/FI/VFI were significantly lower in stimulated cycles. This reduction occurs in approximately 60% of patients after ovarian stimulation. No differences in uterine artery PI and RI between stimulated and spontaneous cycles were found. Neither in stimulated or spontaneous cycles could a correlation be demonstrated between uterine artery PI/RI and any subendometrial/endometrial 3D-PDA indices and between serum estradiol levels and 3D-PDA indices (r value ranging from 0.04 to 0.36). A moderate correlation between endometrial and subendometrial VI/FI/VFI in stimulated cycles and natural cycles within the same patients was found. It was rather surprising the lack of correlation between 3D-PDA indices and serum estradiol levels. The authors could provide no clear explanation for this finding.

In a subsequent study by the same group, including a larger series, a significant but weak negative correlation between uterine artery PI/RI and subendometrial 3D-PDA indices in both stimulated and natural cycles was reported (r values ranging from -0.14 to -0.31). Uterine artery RI was negatively correlated with endometrial VI and FI in natural cycles. These authors concluded that uterine blood flow is a poor reflection of subendometrial vascularization during stimulated and natural cycles and cannot reflect endometrial blood flow [53].

Different dose of recombinant hCG (250 μg/day vs 500 μg/day) for ovarian stimulation does not affect endometrial and subendometrial blood flow [54]. However, the same group reported that endometrial and subendometrial VI/VFI on hCG +2 day in excessive responders (estradiol levels > 20000 pmol/L after long protocol of pituitary down regulation) tended to be lower than moderate responders (estradiol levels < 20000 pmol/L), whereas endometrial FI and subendometrial VFI tended to be higher in day hCG +7 [55].

Raine-Fenning et al found that smoking was related to lower subendometrial VI and VFI, whereas subendometrial FI was higher in parous women. No differences were found regarding parity or smoking habits in endometrial vascularization [46]. On the other hand, endometrial and subendometrial vascularity during the mid and late follicular phase was found to be significantly reduced in women with unexplained subfertility, irrespective of estradiol and progesterone levels [56].

However, Ng et al found that women age, smoking, type of infertility and cause of infertility had no effect on endometrial and subendometrial 3D-PDA indices [57]. Once again, these controversial results could be explained by different population, study design and methods used.

Small uterine intramural fibroids and the presence of unilateral or bilateral hidrosalpinges do not affect endometrial and subendometrial blood flow as assessed by 3D-PDA [58,59].

These studies are summarized in table 2.

**Table 2:** Summary of data published about factor that may affect 3D-ultrasound assessment of menstrual cycle

Author	n	Primary Outcome	3D Method	Study Design	Findings
Ng (52)	67	Endometrial and subendometrial vascularity in spontaneous and stimulated cycles	VOCAL	Cross-sectional: oocyte retrieval day in stimulated cycles and LH surge day in spontaneous cycles	Endometrial and subendometrial vascularity was significantly lower in stimulated cycles as compared with spontaneous cycles
Ng (53)	645	Endometrial and subendometrial vascularity in spontaneous and stimulated cycles	VOCAL	Cross-sectional: oocyte retrieval day in stimulated cycles and LH surge day in spontaneous cycles	Uterine PI and RI were weakly correlated with endometrial and subendometrial VI/FI/VFI, both in spontaneous and stimulated cycles.
Chan (54)	60	Endometrial volume, and endometrial and subendometrial vascularity	VOCAL	Cross-sectional: oocyte retrieval day	Endometrial and subendometrial VI/FI/VFI are not affected by different r-hCG dosage
Ng (55)	32	Endometrial and subendometrial vascularity in stimulated cycles	VOCAL	Longitudinal hCG +2, hCG +4, hCG +7	Changes in endometrial and subendometrial VI/FI/VFI are different in excessive responders as compared with moderate responders.
Raine-Fenning (56)	48	Endometrial and subendometrial vascularity in fertile and unexplained subfertile women	VOCAL	Longitudinal at 2 days interval in follicular phase and 4 days interval in luteal phase	Endometrial and subendometrial VI/FI/VFI were significantly lower in women with unexplained subfertility
Ng (57–59)	645	Endometrial and subendometrial vascularity	VOCAL	Cross-sectional Oocyte retrieval	Endometrial and subendometrial VI/FI/VFI are not affected by women's age, smoking, type and cause of infertility, presence of hydrosalpinx or uterine fibroids

## 3D ultrasound for predicting endometrial receptivity in ARTs

The term "uterine receptivity" refers to a state when endometrium allows a blastocyst to attach, penetrate and induce changes in the stroma, which results in the so-called process of implantation. It appears that a favourable endometrial milieu in necessary for successful implantation and, although various endocrine parameters correlated with endometrial receptivity and implantation are well-documented [60-62] what determines such a favourable milieu, however, is still poorly understood.

The standard method of endometrial dating is the histological evaluation of an endometrial biopsy [1]. Obviously, such an invasive method is not acceptable in order not to damage the endometrium. Therefore, endometrial receptivity should be ideally assessed before embryo transfer using a non-invasive method.

Transvaginal ultrasonography may represent theoretically such an ideal non-invasive technique. Several sonographic parameters have been used to assess uterine receptivity, including endometrial thickness, endometrial pattern and endometrial subendometrial and uterine blood flow [4,5,63]. However, many studies performed in the last 15 years clearly show that all of these sonographic parameters have a low predictive value for determining endometrial or uterine receptivity [8]. Therefore, the method to predict endometrial receptivity has yet to be established.

With the advent of three-dimensional ultrasound it became possible to perform a reliable and reproducible sonographic endometrial volume calculations as well as an assessment of endometrial and subendometrial vascularization. Therefore, some researchers have evaluated the role of endometrial volume as well as subendometrial and endometrial vascularization for predicting uterine receptivity.

Regarding endometrial volume, most studies published to date conclude that endometrial volume does not predict endometrial receptivity (Table 3).

**Table 3:** Summary of data published about the role of 3D-ultrasound for predicting outcome in IVF program

Author	N	Primary outcome	3D Method	Day 3D US	Sub endometrial area	Pregnancy rate (PR)	Findings
Raga (65)	72	Pregnancy rate	Multislice	Embryo transfer		29.2%	No pregnancy if endometrial volume < 1 ml If endometrial volume ≥ 2 ml, no difference in PR
Schild (64)	47	Pregnancy rate	Multislice	Oocyte retrieval		31.9%	No difference in endometrial volume between conception and non-conception cycles
Yaman (66)	65	Pregnancy rate	Multislice	HCG		32.3%	No difference in endometrial volume between conception and non-conception cycles No pregnancy if endometrial volume < 2.5 ml
Zollner (67)	125	Pregnancy rate	Multislice	Embryo transfer		27.2%	PR 35% if endometrial volume ≥ 2.5 ml PR 9% if endometrial volume < 2.5 ml
Schild (68)	96	Pregnancy rate	Multislice	1^st ^day ovarian stimulation		20%	Subendometrial VI, FI and VFI lower in conception cycles
Kupesic (69)	89	Pregnancy rate	Multislice	Embryo transfer	5 mm	31.5%	No difference in endometrial volume, subendometrial VI and VFI between conception and non-conception cycles Subendometrial FI higher in conception cycles
Wu (79)	54	Pregnancy rate	Multislice	HCG	5 mm	50%	Subendometrial VFI higher in conception cycles. No differences in subendometrial VI and FI No differences in endometrial VI, FI and VFI
Jarvela (71)	35	Pregnancy rate	VOCAL	Before HCG and 36 hours after oocyte retrieval	10 mm	37%	No difference in endometrial volume, endometrial/subendometrial VI. FI and VFI
Ng (73)	451	Pregnancy rate	VOCAL	Oocyte retrieval	1 mm	20.8%	Endometrial VI and VFI lower in conception cycles. No differences in endometrial volume and subendometrial VI, FI and VFI

Schild was the first to correlate endometrial volume and pregnancy rate is an IVF program. These authors evaluated 47 patients using the multi-slice technique for endometrial volume calculation. Ultrasound examination was performed on the day of oocyte retrieval (36 h after hCG administration) after pituitary down regulation protocol. Pregnancy rate was 31.9% (15/47). They found that endometrial volume failed to predict outcome of IVF and that estradiol levels did not correlate with endometrial volume [64].

Almost simultaneously, Raga reported on 72 patients who underwent IVF cycle. These authors used the same technique than Schild for calculating endometrial volume but ultrasound examination was performed on the day of embryo transfer (48 h after oocyte retrieval). Pregnancy rate was 29.2%. These authors found that pregnancy rate was significantly lower (15%) if endometrial volume was < 2 ml than if it was > 2 ml (34.5%). No pregnancy was achieved with endometrial volume below 1 ml [65]

Yaman reported subsequently in 65 patients undergoing IVF program [47]. The 3D-ultrasound technique was similar than in previous studies, but performed on the day of HCG administration (48 h prior to oocyte retrieval and 96 h prior to embryo transfer). Pregnancy rate was 32.3%. They found that endometrial volume did not differ significantly in women that became pregnant from those who did not. No pregnancy occurred of endometrial volume was < 2.5 ml. However, the specificity of endometrial volume was so low that it lacked of clinical value.

Zollner evaluated endometrial volume in 125 women undergoing IVF [66]. Pregnancy rate was 27.2%. They found that pregnancy rate was lower in patients with endometrial volume < 2.5 ml (9.4%) compared with those with endometrial volume ≥ 2.5 ml (35%). However, again these findings lacked of specificity.

All studies more recently published did not find differences in endometrial volume between those patients who became pregnant and those who did not after IVF program [68-72]

Angiogenesis plays a critical role in various female reproductive processes such as development of a dominant follicle, formation of corpus luteum, endometrial growth and implantation [42-44]. For this reason many researches have paid attention to ovarian and uterine/endometrial vascularization for predicting outcome in IVF programs [73].

Conventionally, pulsed and colour Doppler have been used to assess uterine and endometrial blood flow. However, conflicting results have been reported. While some authors [69] have found that spiral artery PI was significantly lower in pregnant cycles as compared with non-pregnant cycles, others have found no differences [74]. Similarly, some authors have pointed out that uterine artery RI or PI are similar in non-conception and conception cycles of patients undergoing similar ovarian stimulation protocols after pituitary down regulation [73].

Three-dimensional power-Doppler angiography (3D-PDA) allows quantitative assessment of vessel density and blood flow within the endometrium and subendometrial region.

Schild evaluated 96 patients undergoing IVF program by 3D-PDA [68]. Ultrasound examination was performed on the first day of ovarian stimulation after pituitary down regulation. Pregnancy rate was 20%. Only subendometrial vascularization was assessed, but the authors provided no definition of "subendometrial region". They found that all 3D-PDA indices were significantly lower in conception with non-conception cycles. However, a great overlapping existed. These findings were the same in a subgroup of patients in which at least two good quality embryos were transferred. Logistic regression revealed that subendometrial FI was the strongest predicting factor of IVF success. No association between uterine artery PI and PSV and IVF outcome was found.

On the other hand, Raine-Fenning found that endometrial and subendometrial vascularity were significantly reduced in women with unexplained subfertility during the mid-late follicular phase, irrespective of estradiol or progesterone concentrations [56].

Kupesic assessed 89 women by 3D-PDA the day of embryo transfer [69]. Subendometrial region was defined as 5 mm outer from endometrial defined borders. Pregnancy rate was 31.5%. No significant differences were found in subendometrial VI and VPI between conception and non-conception cycles. Subendometrial FI was significantly higher in the pregnancy group. These results were opposite to those reported by Schild [68]. These conflicting results might be explained by the fact of different timing when performing 3D-PDA assessment.

Wu evaluated prospectively 54 patients undergoing IVF-ET [70]. 3D-PDA evaluation was performed on the day of hCG administration. Subendometrial region was defined as 5 mm surrounding the endometrial borders. Pregnancy rate was 50%. They did not find differences on subendometrial VI and FI between conception and non-conception cycles. However, subendometrial VFI was significantly higher in those patients who become pregnant. The best prediction rate was achieved by a subendometrial VFI > 0.24, with a sensitivity of 83.3%, specificity of 88.9%, positive predictive value of 93.8% and negative predictive value of 93.8% and negative predictive value of 72.7%.

Järvelä evaluated endometrial and subendometrial vascularization by 3D PDA in 35 women undergoing IVF [71]. Three-dimensional ultrasound was performed twice, one after FSH stimulation but before hCG administration and a second time the day of oocyte retrieval (36 hours after hCG administration). They used the VOCAL program with a 15°-rotation step. They defined the subendometrial region as the region 10 mm beneath myometrial-endometrial junction. Pregnancy rate was 37%. These authors did not find differences on endometrial and subendometrial vascularization between conception and non-conception cycles. However, they found than in both conception and non-conception cycles endometrial and subendometrial VI decreased significantly between the two examinations this finding would be in agreement with the findings of Raine-Fenning in natural cycles, who reported a decrease of endometrial vascularity during the periovulatory period [46]

More recently, Ng assessed endometrial and subendometrial 3D-PDA indices in 525 women undergoing the first IVF cycle [73]. Ultimately 451 cycles were eligible. Pregnancy rate in this series was 20.8%. They used the vocal program with 15°-rotation step. Subendometrial region was considered to be within 1 mm of the originally defined myometrial-endometrial contour. Ultrasound evaluation was performed on the day of oocyte retrieval. They found that patients in the pregnant group had significantly lower endometrial VI and VFI than those in the non-pregnant group. Endometrial FI, and subendometrial VI, FI and VFI were similar. Multiple logistic regression analysis showed that from multiple parameters only the number of embryos replaced and endometrial VI significantly improved the chance of pregnancy, but this latter had only a marginal predictive value (odd ratio: 0.87, 95% CI: 0.76–0.99).

In this series the authors evaluated a subgroup of patients (n = 166) defined as a good prognosis group (age < 35 years, endometrial thickness > 8 mm, transfer of two or more good quality embryos and the availability of those embryos). There were no differences on 3D-PDA indices between non-pregnant and pregnant groups in this theoretically good prognosis group. These findings are in agreement with those reported by Schild [68] and those from Mercé, who did not find differences in endometrial/subendometrial VI, FI and VFI between pregnant and non-pregnant women when at least two good quality embryos was transferred. However, when first one or no good quality embryos were transferred all three endometrial VI, FI and VFI were significantly higher in those women who became pregnant as compared with those who did not (Mercé LT, personal communication).

Similar results were reported by Ng et al in a subsequent study for frozen-thawed embryo transfer cycles [75].

These data could indicate that endometrial vascularization might be a non-relevant factor when good quality embryos are transferred but could be an important factor when no quality embryos are transferred. These papers are summarised in table 3

## Conclusion

Three-dimensional ultrasound has been proposed as a promising tool for evaluating the endometrium but a review of the literature regarding its role for assessing endometrial function reveals the limitations of this technique.

Endometrial volume has been shown to be ineffective for predicting pregnancy in IVF program in the vast majority of published studies.

Regarding the role of endometrial and subendometrial vascularity assessment the results of several studies are clearly controversial, with some studies finding that endometrial/subendometrial vascularity is increased [69,70] while others found no differences [71]. On the other hand, results are quite different regarding which 3D-PDA index is predictive for pregnancy, for some authors is FI [58] for others is VFI [70] while others established that it was VI [72]

An explanation for these controversial findings might be the different design of reported studies, specially the timing of ultrasound evaluation.

In summary, although 3D ultrasound seems to be a very interesting tool for assessing the endometrium, its current clinical value in predicting pregnancy in IVF should be considered as limited in view of the controversial results published to date.

A consensus about the timing of this technique to be used within an IVF program is needed in order to design new further prospective studies.

## Competing interests

The author declares that there are no financial and non-financial competing interests in relation to this manuscript.

## Authors' contributions

The author contributed to conception, acquisition, analysis and interpretation of the data as well as drafting the manuscript and revising the intellectual content, giving final approval.
